# TSSC: a new deep learning model for accurate pea leaf disease identification

**DOI:** 10.3389/fpls.2025.1718758

**Published:** 2025-12-01

**Authors:** Laixiang Xu, Yibu Chang, Chenyang Li, Yang Zhang, Xiaodong Yang, Xinjia Chen, Zhaopeng Cai, Junmin Zhao

**Affiliations:** 1 School of Computer and Data Science, Henan University of Urban Construction, Pingdingshan, China; 2 Key Laboratory of Biopharmaceutical Preparation and Delivery, Institute of Process Engineering, Chinese Academy of Sciences, Beijing, China; 3 Institute of Food Science and Technology, Chinese Academy of Agricultural Sciences, Beijing, China; 4 Binary Graduate School, Binary University of Management & Entrepreneurship, Puchong, Selangor, Malaysia

**Keywords:** plant pathology, pea leaf, deep learning, convolutional neural network, split attention

## Abstract

**Problem:**

Accurate diagnosis of plant diseases is crucial for ensuring crop yield and food safety. This study aims to explore a deep learning based intelligent recognition methods for plant leaf diseases to solve the automatic recognition problem of various pea leaf diseases.

**Methodology:**

We propose a novel deep learning framework called TSSC. First, a three-neighbor channel attention is designed to promote the effectiveness of feature extraction. Second, a complementary squeeze and excitation mechanism is introduced to enhance the ability to extract key features. Finally, a split attention module is embedded to reduce model complexity.

**Results:**

The experimental results demonstrate that the proposed model achieves an overall classification accuracy of 99.61% and outperforms other excellent deep learning models.

**Contribution:**

The currently proposed system provides an effective solution for image recognition of complex plant diseases and has reference value for the development of mobile disease detection equipment.

## 1 Introduction

Plant pathology mainly studies the pathogens, occurrence, development, and prevention of plant diseases. Leaves are the main parts for identifying plant diseases. By observing the characteristics of leaf lesions (Grabka et al., 2022), we can intuitively determine the type and severity of the disease. When plants are disturbed and exhibit identifiable symptoms, diseases will occur. Plant disease factors mainly include external environmental factors and internal genetic factors (Todd et al., 2022). It is divided into noninfectious diseases and infectious diseases. Plant diseases are the main obstacles to agricultural production (Bhosale et al., 2023). It constrains the high-quality, efficient, and sustainable development of agriculture (Che et al., 2022). At present, it mainly relies on spraying pesticides for prevention and control, but excessive use can easily lead to excessive residues in agricultural products and impose a heavy burden on the environment and human resources (Bhosale et al., 2025). Therefore, accurately and timely assessing the severity of diseases is a prerequisite for achieving precise medication and effective prevention and control.

In recent years, although plant pathology image analysis technology has provided new approaches, it still faces challenges such as complex backgrounds (Deng et al., 2020), high similarity between diseases (Kennedy and Huseth, 2022), and precise segmentation of disease spots. With the continuous development of computer vision technology, deep learning techniques have shown great potential in plant pathology image classification, but existing models still have limitations. For example, as the number of network layers increases, different levels of feature information and differentiation may be lost, which affects the model’s ability to capture subtle disease features. Therefore, developing new methods that can more accurately and efficiently identify plant leaf diseases remains a challenging and important task.

We propose a new deep learning-based pea leaf disease diagnosis model. First, we design a complementary squeeze and excitation module to adaptively allocate weights to feature channels, allowing the model to focus more accurately on key features that can effectively distinguish different diseases, greatly improving the specificity and discriminative power of feature expression. Second, we built a three-neighbor channel attention module to deeply explore the potential relationships between features, which can more sensitively capture the subtle pattern differences of disease features in the channel dimension, enhance the model’s ability to capture complex disease feature patterns, and help distinguish similar disease features. Finally, we introduce the split attention module to further refine the feature processing flow, enabling the model to more accurately distinguish the features of different categories of diseases and improve classification performance. The main contributions of this paper are as follows:

1. We develop a non-dimensional three-neighbor channel attention module that does not take any steps to reduce dimension or associate any channels. This module is designed to pay attention to all channels. It makes the model easier to understand, improves the efficiency with which features are extracted, and helps the model perform better in terms of classification.
2. We propose a complemented squeeze and excitation mechanism, which extracts both the main salient features from the original features and the secondary salient features from the suppressed residual channel. By combining these mutually exclusive features, it is possible to produce a feature representation that is more effective and to improve the capability of feature extraction.
3. We create a split attention module and send the fusion features into the split attention network again. During the process of feature extraction, it further learns local inter-category information that is more nuanced and diverse, it strengthens the close correlation between channels, and it improves the recognition rate of the entire network.

Overall, we propose a pea leaf disease recognition model that includes several attention mechanisms. For the first time, we have implemented the three-neighbor channel attention (TNCA) module, complementary squeezing and excitation (CSE), and split attention (SA) methods in a convolutional neural network (CNN) to enable successful collaborative optimization. We fully leverage the benefits of each attention mechanism in feature channel weight allocation, neighborhood feature association mining, and feature segmentation refinement, thereby significantly enhancing the ability to extract and identify characteristics of pea leaf diseases. In response to the diversity and similarity of visual features of different diseases in pea leaves, we have utilized the synergistic effect of multiple attention mechanisms to accurately capture subtle differences in disease characteristics, significantly improving the accuracy and robustness of pea leaf disease recognition. The proposed model provides a new multi-attention mechanism fusion approach for plant leaf disease recognition, with good scalability and reference value, and is expected to promote the further development of intelligent plant disease recognition technology.

## 2 Literature reviews

The methods for plant pathology image recognition mainly include traditional machine learning and popular deep learning techniques (Xu et al., 2021a). Classic machine learning (Cai et al., 2020a) algorithms such as principal component analysis (PCA) and support vector machines (SVM) are often used in the identification of plant diseases and pests. For example, Zhao et al. (2022) suggested a multi-step strategy based on hyperspectral imaging (Cai et al., 2020b), K-means clustering, SVM, and random forest to discriminate plant stressors. The overall accuracy on the tea green leafhopper, anthracnose, and sunburn was 90.69%. Huang et al. (2022) described an alternaria leaf spot disease forecasting model based on mobile internet disease survey data and high-resolution spatial-temporal meteorological data (Cai and He, 2021). They tested the suggested model using logistic regression, Fisher linear discriminant analysis, SVM, and K-nearest neighbors, yielding an overall accuracy of 88%. Ansari et al. (2022) introduced a new SVM and image processing-enabled approach for detecting and categorizing grape plant leaf diseases with 96% accuracy. Li et al. (2022) proposed using only two wavelength pictures and linear partial least squares discriminant analysis, PCA, Monte Carlo cross-validation, and successive projection techniques to swiftly identify citrus with early decline. It has a classification accuracy of 96.6% overall. Archana et al. (2022) generated an average accuracy of 97.6% using a unique intensity-based color feature extraction method based on the gray-level cooccurrence matrix, bit pattern features, and SVM for rice brown spot, bacterial leaf blight, and blast disease identification. Despite their great classification accuracy, traditional machine learning algorithms rely largely on lesion segmentation and manual design.

In order to overcome the shortcomings of traditional machine learning methods, researchers have turned to excellent deep learning techniques. Deep learning techniques (Devisurya et al., 2022) are increasingly applied in plant pathology image processing (Xu et al., 2021b) and classification as artificial intelligence (Tugrul et al., 2022) develops. Deep learning, which is based on data-driven models, can automatically retrieve the global aspects of plant pathology images without the need for manual design. For example, Sasikaladevi (2022) proposed a deep convolutional neural network model for accurate and rapid identification of plant diseases. Its maximum accuracy was 99.7% on the plant village dataset. Waheed et al. (2022) presented a convolutional neural network model for ginger disease rhizome detection. Its accuracy was 99%. Compared with VGG-16, it improved by 3%. Huang et al. (2023) proposed a fully convolutional switchable normalization dual-path network for automatic identification and detection of tomato leaf diseases. Its accuracy was 97.59%. This is about a 1.52% improvement in classification performance when compared to DenseNet. Russel and Selvaraj (2022) used a specialized parallel multiscale stream with learnable filters based on deep streams of networks for 39 classes of plant pathology leaves and achieved an accuracy of 98.61% on the Plant Village dataset. Adem et al. (2022) suggested an enhanced Yolov4 (Xu et al., 2023) model for automatic detection of sugar beet leaf spot disease and severity classification, with a classification accuracy rate of 96.47%. Pandian et al. (2022) proposed a novel deep residual convolutional neural network with 197 layers that generated an average classification accuracy of 99.58% for the identification of several plant leaf diseases. Feng et al. (2022) reported an average accuracy of 97.8% for an online recognition technique for peanut leaf diseases based on the data balance algorithm, MobileNetV2, and deep transfer learning. Gautam et al. (2022) created a convolutional neural network based on deep learning architecture and a hybrid model for paddy leaf disease classification that achieved 96.4% accuracy.

Although the preceding research thoroughly reveals the great capabilities and enormous promise of deep learning in the field of plant disease recognition, present deep learning models face several significant problems and have room for improvement. As the number of network layers grows, feature information at various levels is readily lost. Deep learning algorithms struggle to adaptively focus on the feature channels that are most important to illnesses, and their capacity to handle complicated correlations and small interclass variations between feature channels needs further improvement. To solve these issues, we present a new deep learning model for accurately diagnosing pea leaf disease. Our key innovation is the integration of the three neighbor channel attention module, complementary squeeze and excitation, and split attention mechanism to achieve collaborative optimization from global feature screening and neighborhood association mining to local feature refinement, which improves the model’s ability to capture and distinguish complex and similar disease features.

## 3 Proposed methods

### 3.1 Three-neighbor channel attention

**Figure 1:**
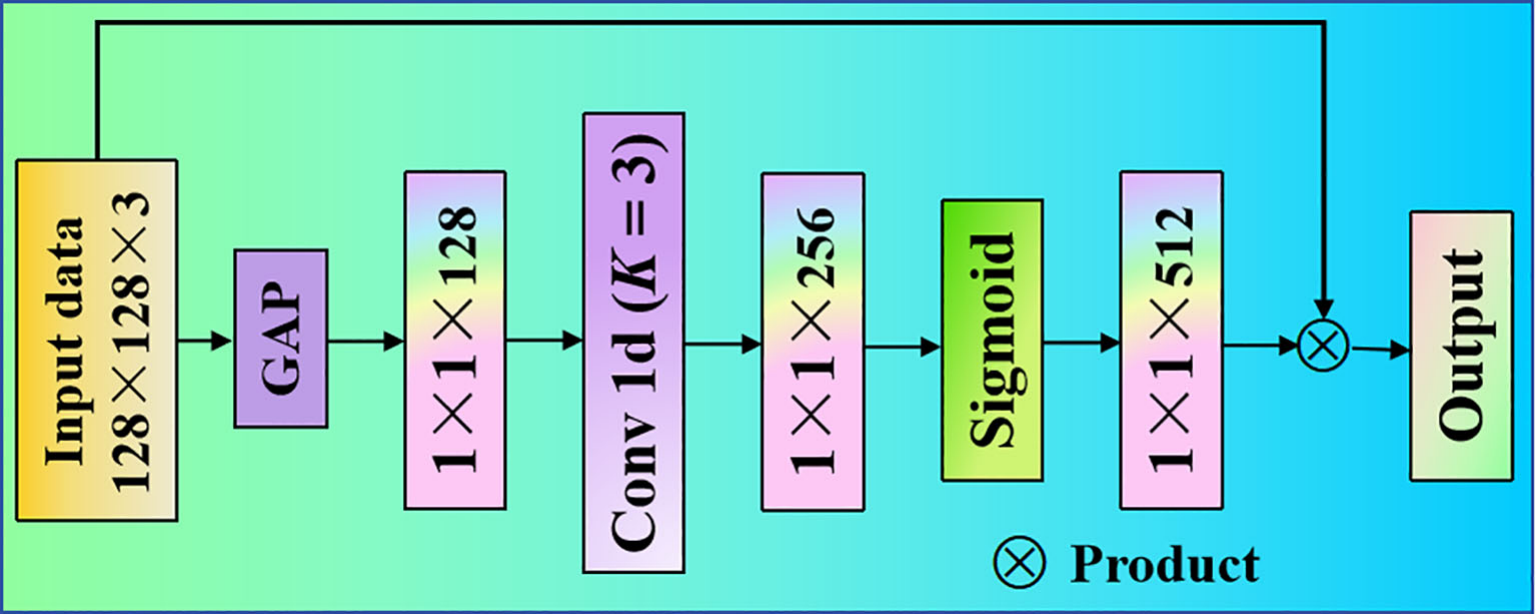
Proposed three-neighbor channel attention module architecture. The GAP is a global average pooling layer. Sigmoid is a activation function.

First, we used two convolutional layers to extract the basic features of pea leaf diseases. After the extraction, we obtain a 128×128×3 feature map, which is input as the proposed TNCA. We send the 128×128×3 feature map into the proposed KNCA for further processing. The proposed TNCA module mainly includes a global average pooling (GAP) layer, a one-dimensional convolution (Conv 1d) layer, and a Sigmoid layer. Then, the proposed TNCA performs a non-dimensional global average pooling operation on each channel of the input block. The output dimension is a feature map of 1×1×128. The one-dimensional convolution operation of a convolution kernel is used to capture the local interaction information between each channel and its three-neighbor channels. The output dimension is a feature map of 1×1×256. The length *K* of the one-dimensional convolution kernel we used is 3. Finally, we use the activate function Sigmoid to output a weight of 1×1×512. The input feature maps and weights are multiplied so that the weights of each channel feature are redistributed. The proposed TNCA is presented in **Figure 1**.

To enhance the model’s ability to capture inter-channel dependencies and spatial context, we introduced a weighting mechanism into the TNCA module. The weighted calculation formula can be defined Equation 1:

(1) $$w=\sigma (f_{\left\{W\right\}}(g(x)))$$

where g(*x*) is the GAP operation, $f_{\left\{W\right\}}$ is the interaction function of a *K*-neighbor channel. σis the activate function Sigmoid. The g(*x*) can be expressed Equation 2:

(2) $$g(x)=\frac{1}{WH}\sum_{i=1,j=1}^{W,H}X_{ij}$$

where $X_{i,j}$ is the pixel value of the feature map at spatial position (i, j). The GAP compresses the two-dimensional feature into a single-value channel descriptor by averaging over the entire spatial dimension of the feature map. W and H represent the width and height of feature maps, respectively. The $f_{\left\{W\right\}}$ can be formulated Equation 3:

(3) $$f_{\left\{W\right\}}=Wg(x)$$

If *y* is *g*(*x*), *w* is $\sigma (W_{y})$. Every channel weight can be calculated Equation 4:

(4) $$w_{i}=\sigma (\sum_{j=1}^{K}w_{i}^{j}y_{i}^{j}),y_{i}^{j}\in \Omega_{i}^{K}$$

where $\Omega_{i}^{K}$ is a set of the channels $y_{i}$ in the proposed TNCA.

During this process, the convolution kernel K can perform one-dimensional convolution operations. TNCA describes weight calculation as the sum of weighted interactions between a channel and its K neighboring channels. This summation operation on adjacent channels is mathematically equivalent to one-dimensional convolution using K-sized convolution kernels. Convolutional kernels perform weighted aggregation within local channel windows. Therefore, *w* can be described Equation 5:

(5) $$w=\sigma (Conv1d_{K}(y))$$

where $Conv1d_{K}$ is one dimensional convolution with the convolution kernel *K. K* can be computed Equation 6:

(6) $$K=N_{in}+2P+1-SN_{out}$$

where $N_{in}$ and $N_{out}$ are the number of input channels and output channels, respectively. To maintain consistency in channel numbering, we set $N_{in}$ = $N_{out}$. The step size S is set to 1.The padding is set to 1. We substitute them into formula (6) to calculate K = 3. It indicates that a one-dimensional convolution kernel captures feature dependencies within the 3-channel neighborhood.

The proposed TNCA module encourages information exchange across channels, thus preventing each feature channel from being treated in isolation. This enhances the overall feature contribution to model recognition and thereby significantly improves classification performance (Zhang et al., 2022).

### 3.2 Squeeze-and-excitation network

The traditional attention mechanism often focuses solely on the most prominent local features, thereby overlooking salient characteristics from diverse semantic components. To address this limitation, we introduce a novel complementary squeeze and excitation mechanism (Hua, 2022). This approach enables the extraction of salient features from various semantic regions within the original feature maps, thereby capturing more discriminative and effective representations.

**Figure 2:**
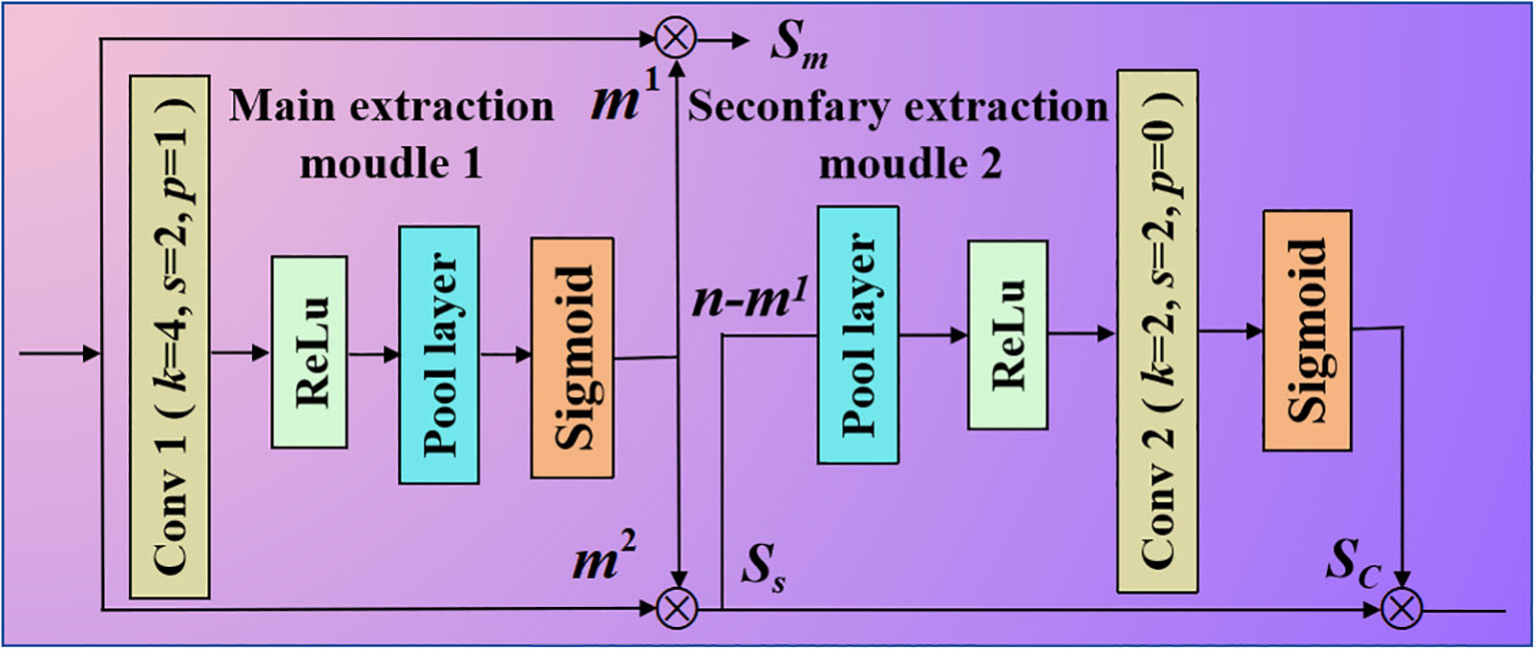
Proposed CSE structure. It mainly consists of a main extraction module 1 and the secondary extraction module 2.

To extract salient features, we used a CSE mechanism to extract the main salient features and secondary salient features, simultaneously. The specific structure is presented in **Figure 2**. *k* is a convolutional kernel, *s* is a stride, and *p* is the padding.

Assume the input feature is $I\in R^{C\times W\times H}$, the characteristic tensor is $U=[u_{1},\cdot \cdot \cdot ,u_{c}]\in R$, $u_{i}$ represents the *i*-dimensional feature. The feature *U* is compressed to generate the channel descriptor $z=[z_{1},\cdot \cdot \cdot ,z_{c}]\in R^{C}$. $z_{c}$ means the *c*-th channel descriptor of *U*. It can be defined Equation 7:

(7) $$z_{c}=\frac{1}{WH}\sum_{w=1}^{W}\sum_{h=1}^{H}u_{c}(w,h)$$

**Figure 3:**
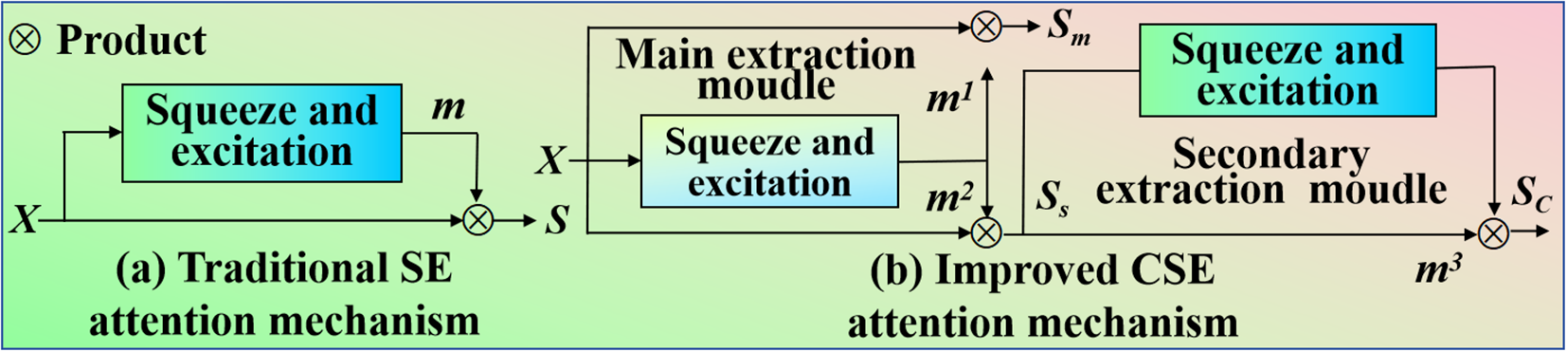
Comparison of two different attention mechanisms. **(a)** is the working principle of traditional squeeze and excitation attention mechanism. **(b)** is the working principle of our improved complementary squeeze and excitation mechanism.

The channel descriptor $z_{c}$ is excited to obtain the corresponding weight vector *m.* Hence, our proposed CSE is different from SE (Liu et al., 2022). The CSE and SE are shown in **Figure 3**.

The proposed CSE not only gets the channel weight vector $m^{1}$ of excitation, but also gets the channel weight vector $m^{2}$ of the suppressed part. *U* outputs two complementary weight vectors $m^{1}$ and $m^{2}$ through exciting, they can be computed Equations 8-9:

(8) $$m^{1}=\sigma (W_{2}\delta (W_{1}z))=[m_{1}^{1},\cdot \cdot \cdot ,m_{c}^{1}]\in R^{C}$$

(9) $$m^{2}=n-m^{1}$$

where σ and δ are Sigmoid and ReLu, respectively. $n\in R^{C}$ represents vectors with all elements of 1. $m^{1}$ and $m^{2}$ are the main significant feature channel weight vector and the suppressed feature channel weight vector, respectively. $W_{1}\in R^{\frac{C}{r}\times C}$is dimension reduction parameters, $W_{2}\in R^{\frac{C}{r}\times C}$ is ascension parameters, *r* represents the multiple of scaling, and we set *r* to 2.

The significant attention features $S_{m}$ and suppressed features $S_{s}$ can be obtained by weighting the channel weights *U. S* can be expressed Equation 10:

(10) $$S=[m_{1}u_{1},\cdot \cdot \cdot ,m_{c}u_{c}]$$

where $u_{c}$ is the *c*-th channel of *u.* $S_{m}$ and $S_{s}$are complementary features of *U. U* is determined Equation 11:

(11) $$U=S_{m}+S_{s}$$

Hence, the salient feature $S_{m}$ is called the main salient feature.

Because the suppressed features $S_{s}$ also contain the effective features, the secondary salient feature extraction module is used to extract the salient features again from the suppressed features $S_{s}$. They can be calculated Equations 12–14:

(12) $$z_{c}^{s}=\frac{1}{WH}\sum_{w=1}^{W}\sum_{h=1}^{H}s_{c}^{s}(w,h)$$

(13) $$m^{3}=s(W_{4}\delta (W_{3}z^{s}))=[m_{1}^{3},\cdot \cdot \cdot ,m_{c}^{3}]\in R^{C}$$

(14) $$S_{c}=[m_{1}^{3}s_{1}^{2},\cdot \cdot \cdot ,m_{c}^{3}s_{c}^{2}]\in R^{W\times H\times C}$$

where $z^{s}$ is a channel descriptor of feature $S_{s}$. $W_{3}\in R^{\frac{C}{r}\times C}$ is dimension reduction parameters, $W_{4}\in R^{\frac{C}{r}\times C}$ is the ascension parameters, *r* is 2. $m^{3}$ represents the weight vectors. $S_{c}$ is called the secondary salient features.

The proposed CSE is able to not only extract the primary salient features from the original features, but it is also able to extract the secondary salient features from the suppression part. Finally, it integrates all of these elements to produce a representation of the desired attributes.

### 3.3 Split attention

**Figure 4:**
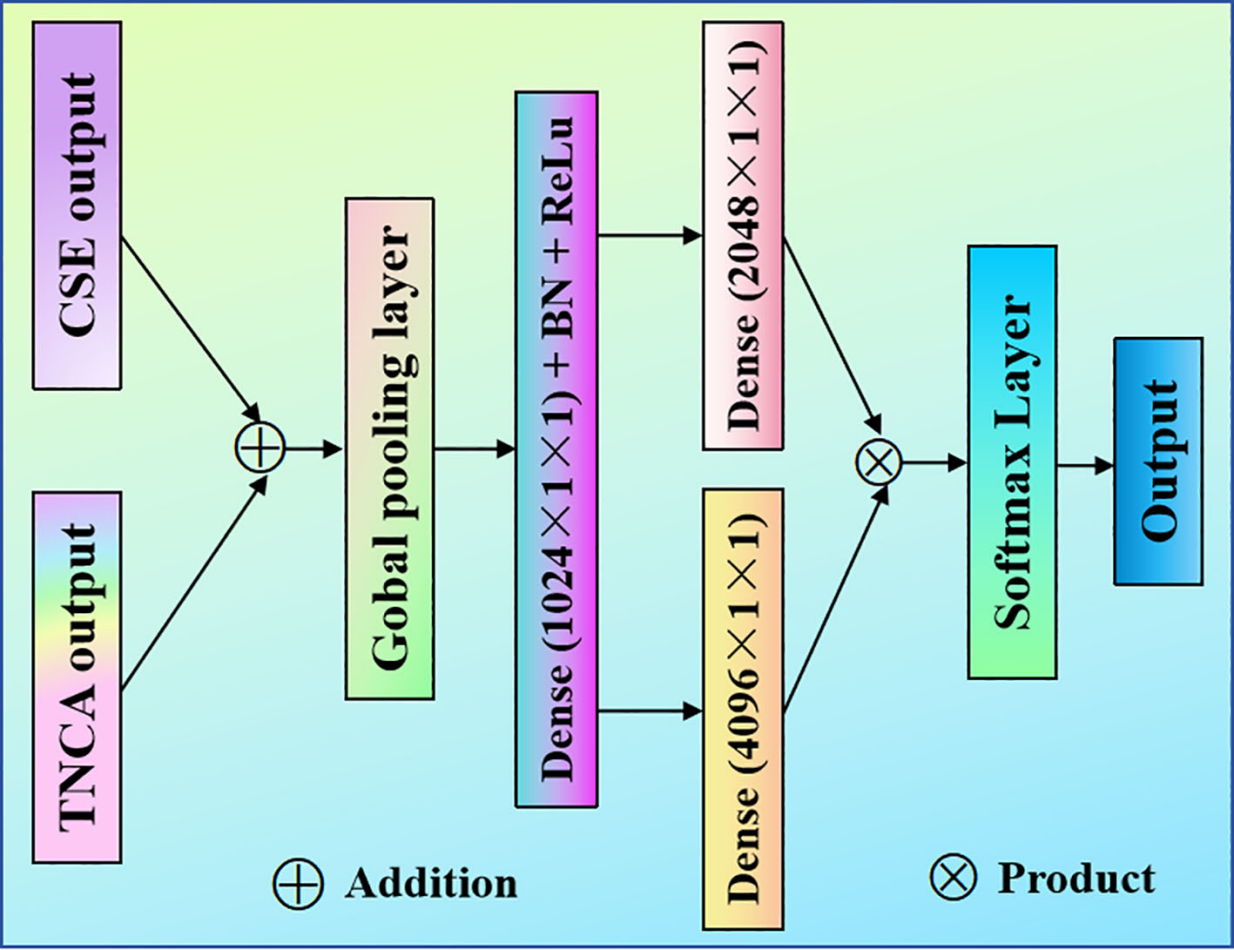
Proposed SA model. The suggested SA module is designed after the TNCA and CSE modules. It is fed into the classifier after multiplying the feature information.

We have implemented a split attention (SA) mechanism in order to cut down on redundant features and compensate for the lack of correlation dependency that exists between feature channels. In feature extraction, we integrate the scattered features and fuse features of different scales. It can enhance the richness of scale features and prevent overfitting. The proposed SA network is sketched in **Figure 4**.

To enhance the model’s focus on spatially critical regions, the proposed spatial attention module leverages densely connected layers (DCL) to adaptively weight spatial features. The process of feature aggregation and dimension manipulation in the SA is described as follows.

Each branch is used to fuse spatial dimensions through a GAP layer. The global context information of each dimension feature graph is compressed into a single channel description by the channel statistics strategy. The channel space dimension *H*×*W* becomes 1×1, and the process is Equation 15:

(15) $$s_{c}=F_{gp}(U_{c})=\frac{1}{H\times W}\sum_{i=1}^{H}\sum_{j=1}^{W}U_{c}(i,j)$$

where c is an index of the channel in the spatial feature map. $U_{c}$ denotes the feature map of the c-th channel, and $F_{Rj}$ represents Global Average Pooling. This operation compresses the spatial dimensions *H*×*W* of each channel into a single scalar, capturing the global context of that channel. It calculates a channel-wise shrinkage factor through the feature map $U_{c}$.

The proposed SA has two densely connected layers (DCL) (Zhang et al., 2020). First, according to the first layer of the DCL, the dimension reduction operation is carried out at the deceleration rate of 1/*r*, and we set *r* to 2. It can obtain more compact feature vectors *W.* We used batch normalization and the ReLu layer to perform *W.* The process can be expressed Equation 16:

(16) $$z=F_{dc}(s)=\delta (ℜ(W_{s}))$$

Hence, *d* is max(C/r,L). δ is ReLu and ℜ is a BN.

The second layer of the proposed DCL has been restored to the dimension. We use the *Softmax* layer to obtain classification probabilities.

Compared to a single DCL, our proposed double DCL incorporates additional nonlinear operations, enhancing its ability to model complex interdependencies between channels. This design also streamlines the computational process, significantly reducing both operational complexity and the number of parameters.

**Figure 5:**
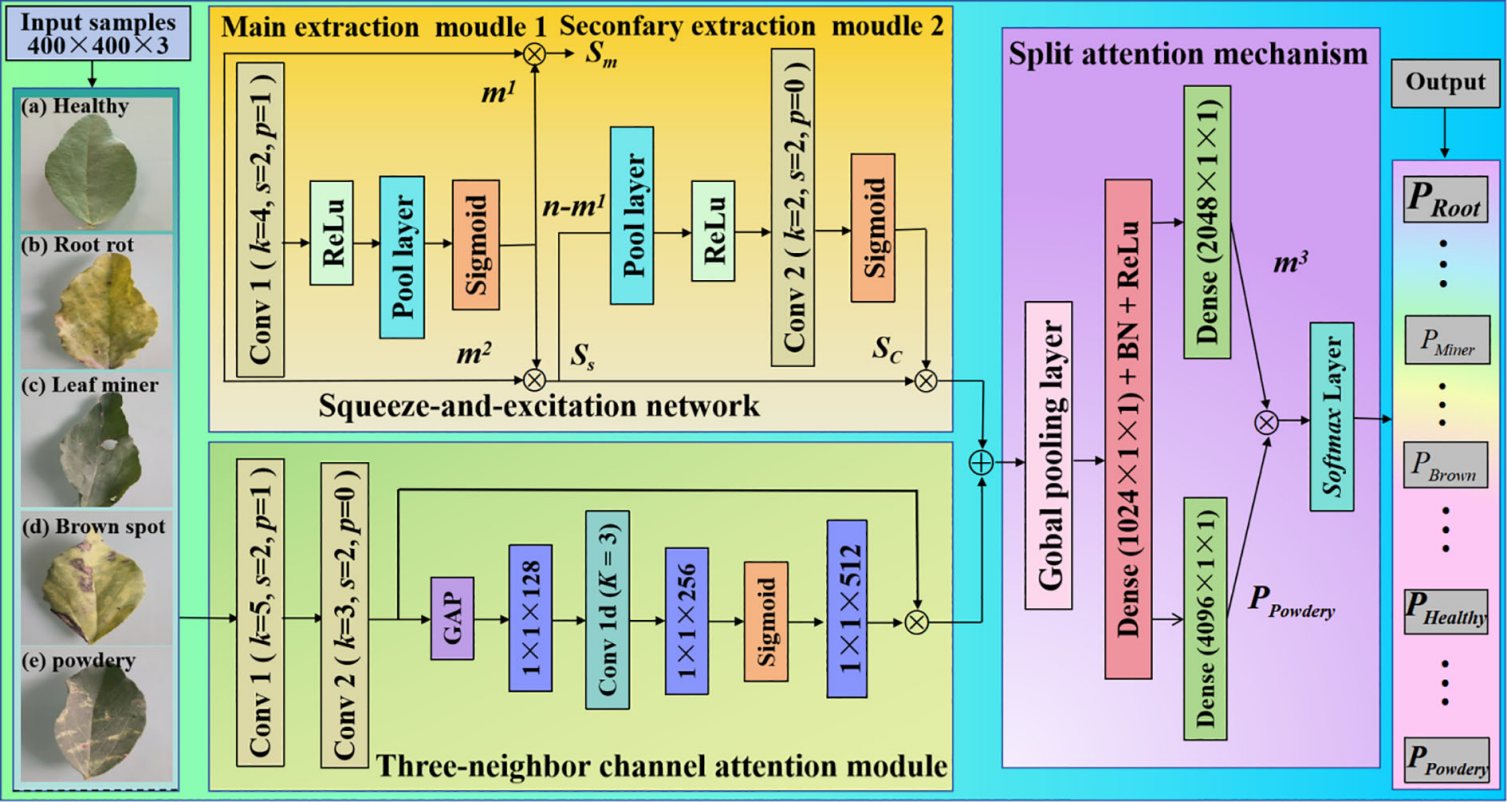
Proposed overall identification framework. It mainly consists of the proposed basic model convolutional neural network, three-neighbor channel attention modules, complementary squeeze and excitation, and a split attention mechanism module.

To intuitively display the overall pea leaf disease identification framework, we present it in **Figure 5**.

The processing pipeline operates as follows: First, both healthy and diseased pea leaf samples are processed through squeeze-and-excitation networks and three-neighbor channel attention modules for preliminary feature extraction. The resulting features are then fed into a split attention module for further integration. Finally, the refined features are passed to a Softmax classifier to generate category predictions and classification scores.

## 4 Experimental results and analysis

### 4.1 Experimental setup

The models are achieved on TensorFlow-gpu1.14.0 and Keras 2.2.4. We use the neural network acceleration library CUDA 9.2 and cuDNN 7.0 to improve training efficiency. The programs run on Anaconda 3 5.2.0 and Spyder 3.3.3. Their programming language is Python 3.10. The operating system is Windows 10× 64-bit. The hardware platform is a 10-core Intel Core i7–9700 CPU@3.00 GHz processor and an NVIDIA GeForce GTX 1080 graphics card for accelerating.

In order to determine the principle of parameter values that perform well in the model, we designed a systematic parameter tuning experiment. During the experiment, we recorded in detail the training dynamics and validation results of the model under each parameter setting at 1000 epochs. These comparative data clearly demonstrate the correlation between parameter selection and model performance. The complete experimental results are presented in **Table 1**.

**Table 1:** Experimental parameter configuration.

Hyperparameter type	Candidate value	Test accuracy	Test loss
Conv Kernel Size	3×3	99.61%	0.0217
Conv Kernel Size	5×5	98.12%	0.0325
Stride	1	99.61%	0.0217
Stride	2	97.35%	0.0542
Padding	Same Padding	99.61%	0.0217
Padding	Valid Padding	98.23%	0.0311
Scaling Factor (SE)	2	99.61%	0.0217
Scaling Factor (SE)	4	96.89%	0.0438
Scaling Factor (TNCA)	1	99.61%	0.0217
Scaling Factor (TNCA)	2	99.32%	0.0222
Dense Layer Neurons	2048	98.57%	0.0217
Dense Layer Neurons	4096	99.61%	0.0212

From **Table 1**, it can be seen that when the convolution kernel size, stride, padding method, scaling factors of the SE and TNCA modules, and the number of dense layer neurons are 3×3, 1, same padding, 2, 1, and 4096, respectively. The model showed good testing accuracy and loss performance. Therefore, we set the convolution kernel size, stride, padding method, scaling factors of the SE and TNCA modules, and the number of dense layer neurons to 3×3, 1, same padding, 2, 1, and 4096, respectively.

### 4.2 Construction of the dataset

**Figure 6:**
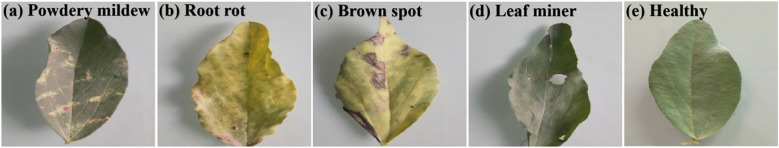
Five categories of plant samples. **(a)** usually presents light yellow spots and irregular pink spots. **(b)** is spread by germs through the soil to the seeds and roots. **(c)** appears light purple spots on the infected leaves. **(d)** is mainly caused by leaf insects. **(e)** keeps uniform and consistent color.

The experimental data came from pea-growing bases in Henan Province, China. The experimental dataset is collected under standardized laboratory conditions (fixed lighting, solid color background, vertical elevation angle), which can effectively control irrelevant interference factors and ensure the pertinence and stability of feature learning during model training. This provides reliable data support for verifying the basic performance of the model that recognizes pea leaf diseases. We used the mobile phone camera to take the experimental data in a laboratory environment. There were a total of 7750 samples, which included healthy leaves as well as four forms of diseases: root rot, leaf miner, powdery mildew, and brown spot. The five samples are presented in **Figure 6**.

According to the data division principle of most deep learning models, the 7750 samples were randomly divided into a training set, a validation set, and a testing set with a ratio of 3:1:1. **Table 2** lists the detailed quantity distributions.

**Table 2:** The division of each category.

Disease categories	Train set	Validate set	Test set	Total number
Root rot	985	328	329	1642
Leaf miner	916	305	305	1526
Powdery mildew	792	264	264	1320
Brown spot	1031	343	343	1717
Healthy	927	309	309	1545
Total number	4651	1549	1550	7750

At present, we are limited by the collection scenarios and conditions. The experimental dataset still has room for expansion in terms of environmental diversity and scene coverage. In the future, we will focus on addressing the following shortcomings: (1) Environmental factor bias: Existing datasets lack samples in natural field environments. It is a model trained solely on laboratory data. When deployed in actual agricultural scenarios, it may face generalization challenges. (2) Sample scenario singularity: The current dataset only covers samples of specific growth stages and leaf positions of peas. It does not cover the diversity of leaf growth stages and positions. This may limit the model’s ability to identify leaf diseases in different physiological states. (3) Limitations in adaptability of collection devices: Existing datasets are collected by fixed high-resolution mobile cameras. In practical applications, farmers or field detection systems may use devices with different imaging qualities. The dataset lacks corresponding cross-device validation samples.

### 4.3 Experimental results

**Figure 7:**
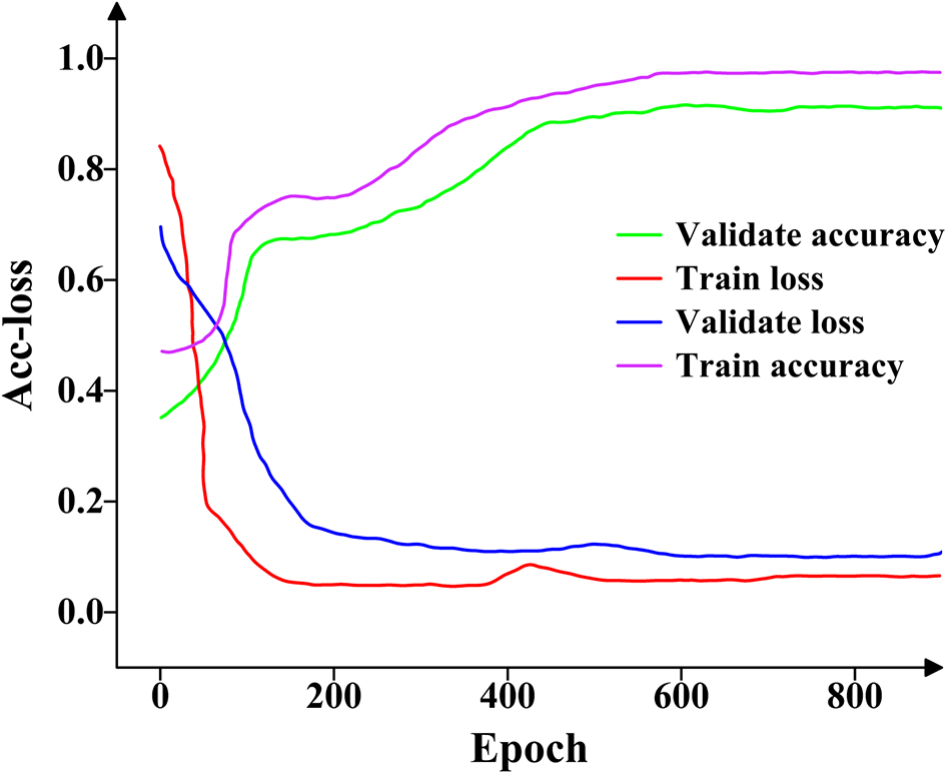
The accuracy and loss change curves on the training and validation datasets. The four curves reflect the learning effect of the proposed model on feature extraction.

The proposed model was trained using the training and validation sets. The accuracy and loss (Acc-loss) values represent the accuracy and loss levels at distinct epochs. **Figure 7** depicts the variations in the loss and accuracy values.

As shown in **Figure 7**, both the training and validation accuracy curves increase gradually between epochs 0 and 600, beyond which they stabilize and saturate without significant fluctuations. Similarly, the training and validation loss curves decrease steadily from epoch 0 to 500, after which they converge and remain stable with no noticeable oscillations. Overall, the proposed model demonstrates strong feature extraction capability for pea leaf diseases.

To test several deep learning models for plant leaf disease identification, we employed specificity, precision, recall, the f1-score, and accuracy. These evaluation metrics can be expressed Equations 17–21:

(17) $$\text{Specificity}=\frac{TN}{TN+FP}$$

(18) $$\text{Precision}=\frac{TP}{TP+FP}$$

(19) $$\text{Recall}=\frac{TP}{TP+FN}$$

(20) $$F1-score=\frac{2\times \text{Precision}\times \text{Recall}}{\text{Precision}+\text{Recall}}$$

(21) $$\text{Accuracy}=\frac{TP+TN}{TP+TN+FP+FN}$$

where true positives (TP), true negatives (TN), false positives (FP), and false negatives (FN) are represented.

**Figure 8:**
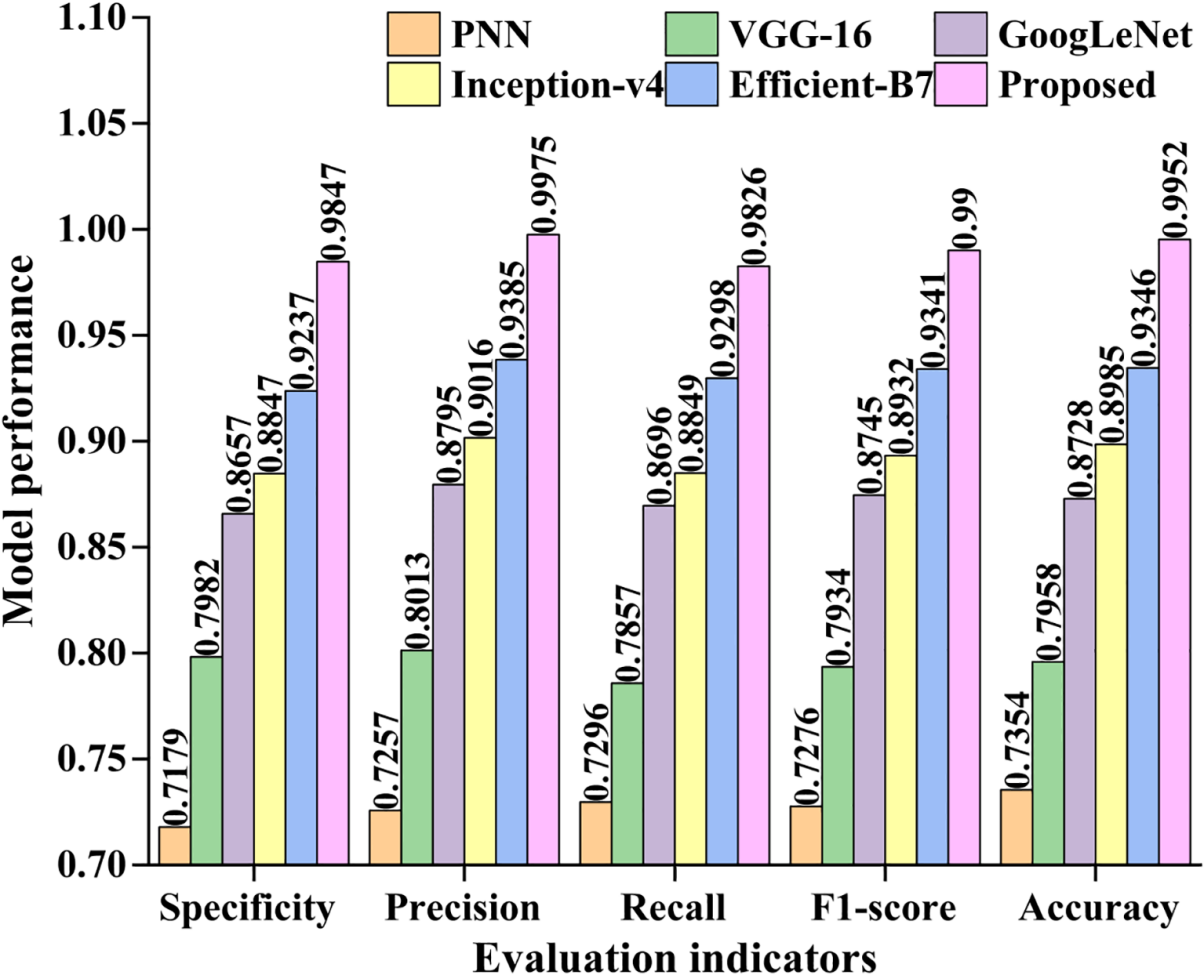
Comparisons of the six different models. It reflects the classification and recognition performance of six different deep learning algorithms on pea leaf diseases under five evaluation indicators.

To test specificity, precision, recall, the f1-score, and accuracy with our dataset, we employed some great deep learning models, including PNN, VGG-16, GoogLeNet, Inception-v4, Efficient-B7, and the suggested model. **Figure 8** displays the testing results for various networks.

**Figure 8** demonstrates that the suggested model outperforms the other five models in terms of specificity, precision, recall, the f1-score, and accuracy. The classification performance of GoogLeNet and Inception-v4 is not significantly different, but they are both lower than Efficient-B7. On the five evaluation indicators, the suggested model outperforms the lowest PNN by roughly 26%. Taking all aspects into account, the suggested model has demonstrated some potential for diagnosing pea leaf diseases.

**Figure 9:**
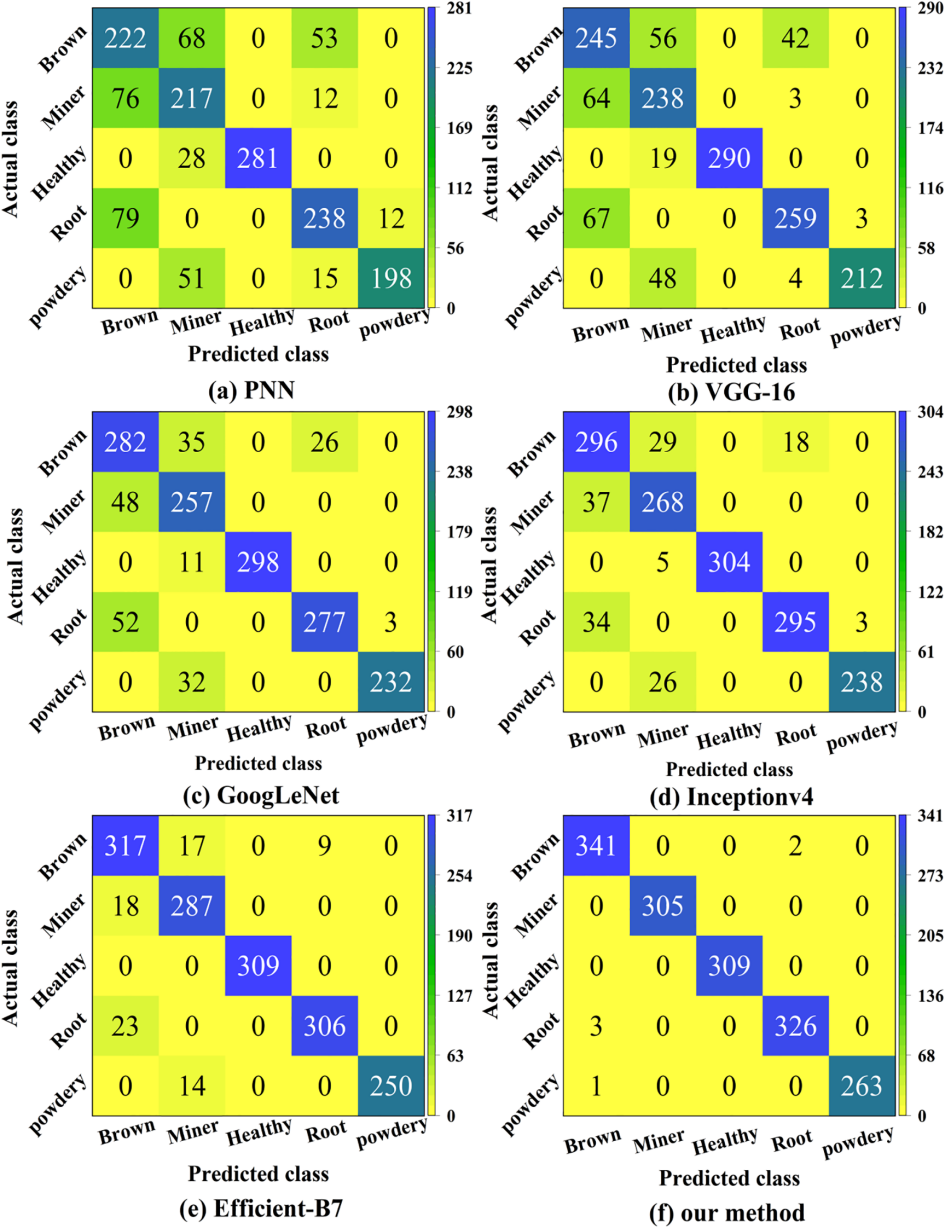
Confusion matrices of the six methods. It reflects the specific distribution of correct and incorrect classification and recognition of five pea leaf diseases by six different deep learning models.

We assessed the accuracy of both the correct and wrong classifications using confusion matrices (Hinterreiter et al., 2020). **Figure 9** presents the classification results of confusion matrices on the test set.

From **Figure 9** we can compute that the overall classification accuracies of the PNN, VGG-16, GoogLeNet, Inception-v4, EfficientNet-B7, and the proposed models are 74.58%, 80.26%, 86.84%, 90.39%, 94.77%, and 99.61%, respectively. Compared to the lowest-performing model PNN, the proposed model shows an improvement of 25.03%. It also outperforms the previously highest-performing model EfficientNet-B7, by 4.84%. The proposed model performs well in classifying leaf miners and powdery mildews, suggesting significant differences in their contour and color features, which facilitates accurate distinction. However, its classification ability is weaker for brown spot and root rot, likely due to the similarity in their color and texture features, leading to a higher likelihood of misclassification. In summary, the proposed model demonstrates high effectiveness in identifying pea leaf diseases.

**Figure 10:**
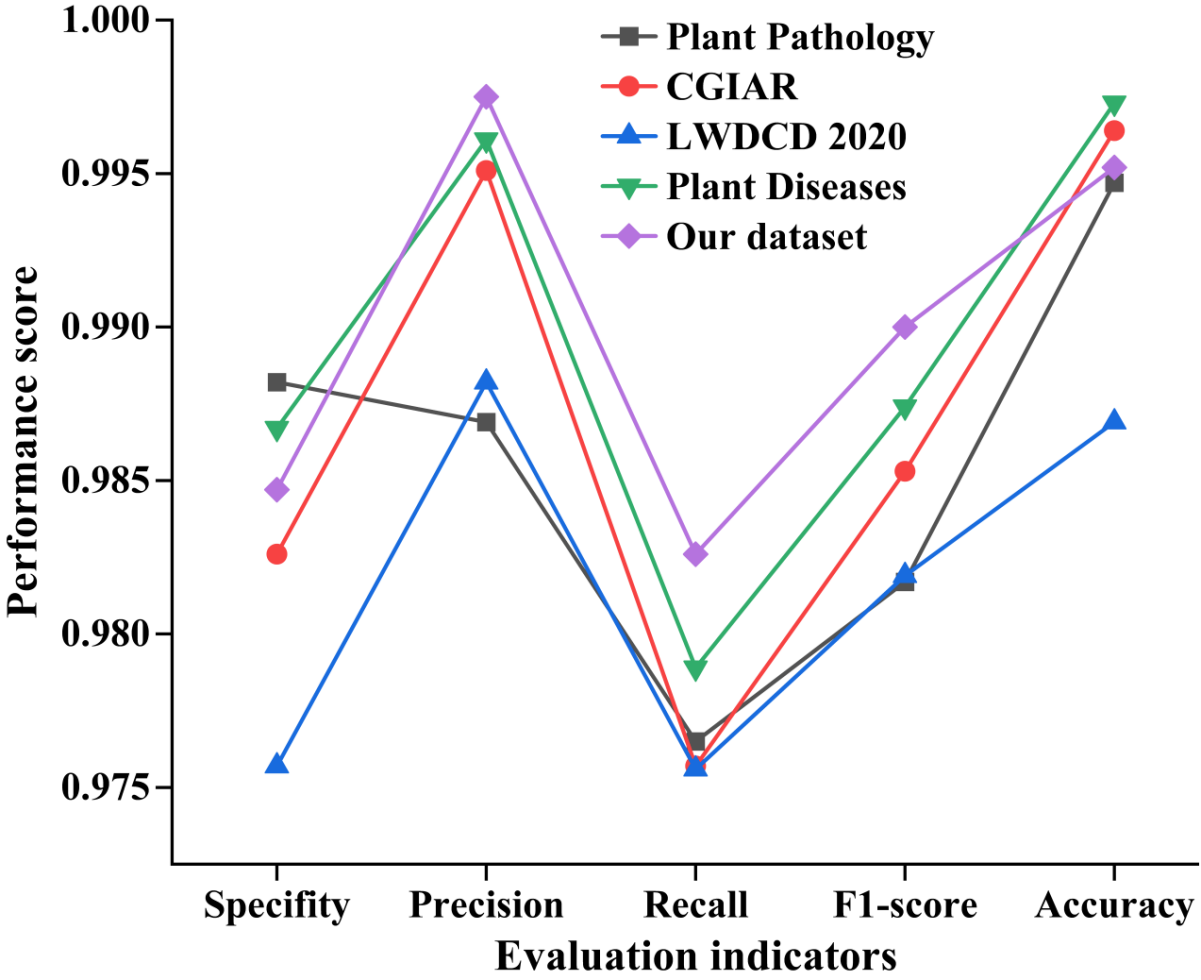
Comparisons of the five different datasets with the proposed method. It reflects the classification and recognition performance differences of the proposed model on five different plant leaf disease data using five performance evaluation indicators.

We used the proposed method to test other open-source datasets. The results are presented in **Figure 10**.

The information presented in **Figure 10** enables us to draw several significant conclusions, one of which is that the proposed method performs better on the other four open-source datasets. The proposed model is higher than the databases, such as Plant Pathology, CGIAR, LWDCD, and Plant Diseases, in terms of the precision, recall, and f1-score. The results of the most recent tests demonstrate that the proposed system has a high degree of generalizability and good feasibility for plant leaf disease identification.

**Figure 11:**
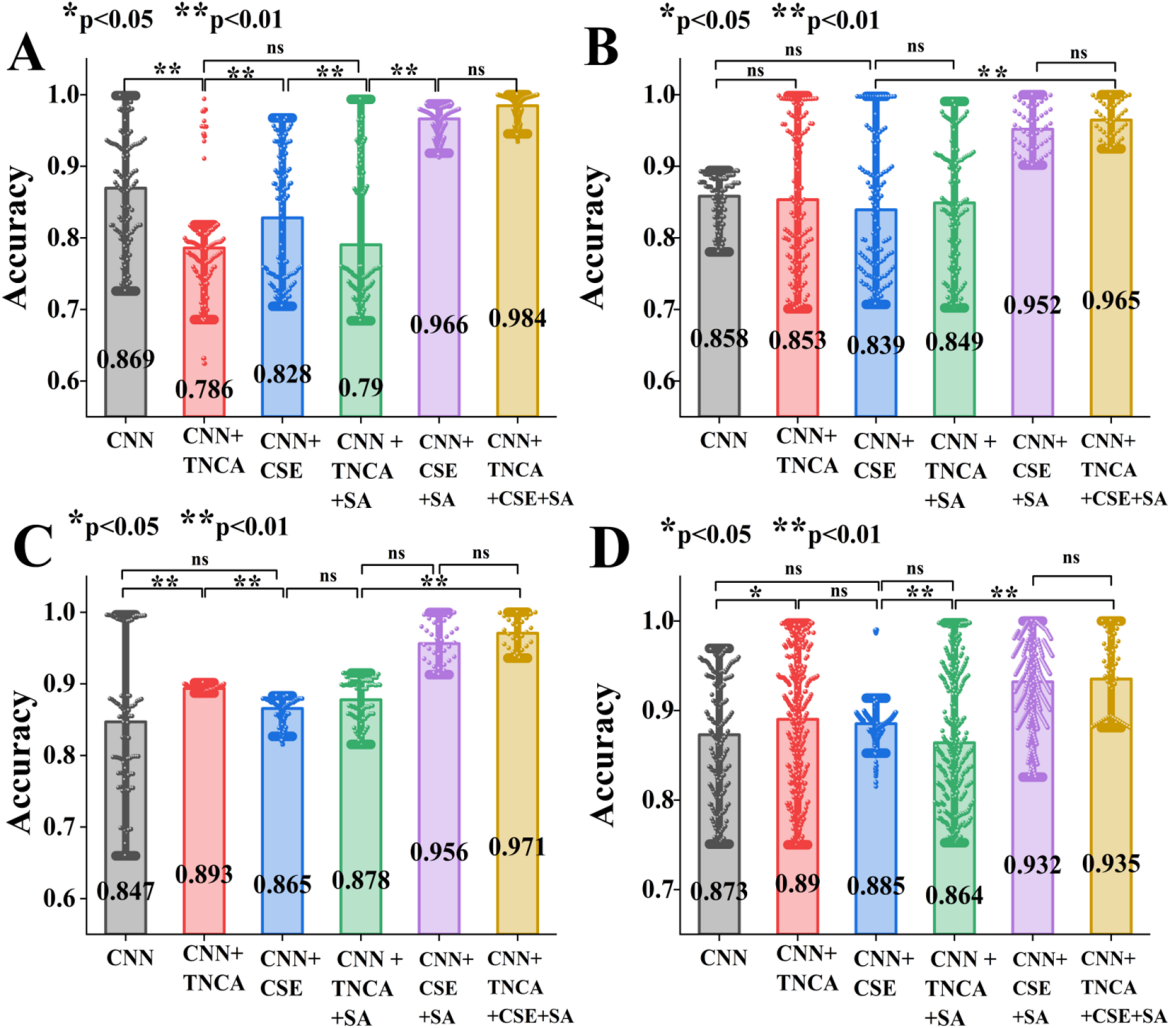
Significant difference bar scatter plot in the ablation experiment of four pea leaf diseases. **(a)** is the root rot disease. **(b)** is the Leaf miner disease. **(c)** is the powdery mildew disease. **(d)** is the brown spot disease.

In ablation experiments, a bar scatter plot with significant differences is a core visualization tool for verifying the effectiveness of each module and quantifying its contribution. It can visually compare the average performance gap between the base model and models that introduce different modules on the target task. Meanwhile, random fluctuation interference is eliminated from the dimensions of stability and statistical reliability using scatter distribution, error bars, and significance markers, allowing for an accurate determination of whether the module’s performance improvement is practical. **Figure 11** depicts bar scatter plots demonstrating significant differences between different models for four diseases. The bar chart’s height represents the average recognition accuracy of different models, while the data scatter distribution reflects the degree of dispersion of sample predicted values. The error bar (95% confidence interval) indicates the statistical fluctuation range of the results, and the asterisk markers (* p<0.05, ** p<0.01) indicate the statistical significance level of performance differences between models.

From **Figure 11**, it can be seen that for the root rot diseases, the proposed model of integrating multiple modules CNN+TNCA+CSE+SA has an accuracy improvement of about 11.5% compared to the basic CNN model. Its statistical test shows a very significant difference. On the leaf miner diseases, adding the TNCA to a CNN alone increases efficiency by 1.4% compared to adding CSE. This indicates that adding the TNCA to the CNN+SA has higher classification performance for identifying leaf miners than adding CSE. On the powdered mildew diseases, adding the CSE to a CNN alone resulted in a 7.8% increase compared to adding the TNCA. This indicates that adding the CSE to the CNN+SA has higher classification performance for identifying the powdered mildew diseases than adding the TNCA. The proposed model of CNN+TNCA+CSE+SA has an accuracy improvement of 6.2% compared to the basic CNN. On the brown spot, the proposed model CNN+TNCA+CSE+SA has an accuracy improvement of 6.2% compared to the baseline CNN. Specifically, the proposed ablation combination models do not conflict with each other. As a consequence, the proposed TSSC model has a high performance advantage in identifying four pea leaf diseases.

**Figure 12:**
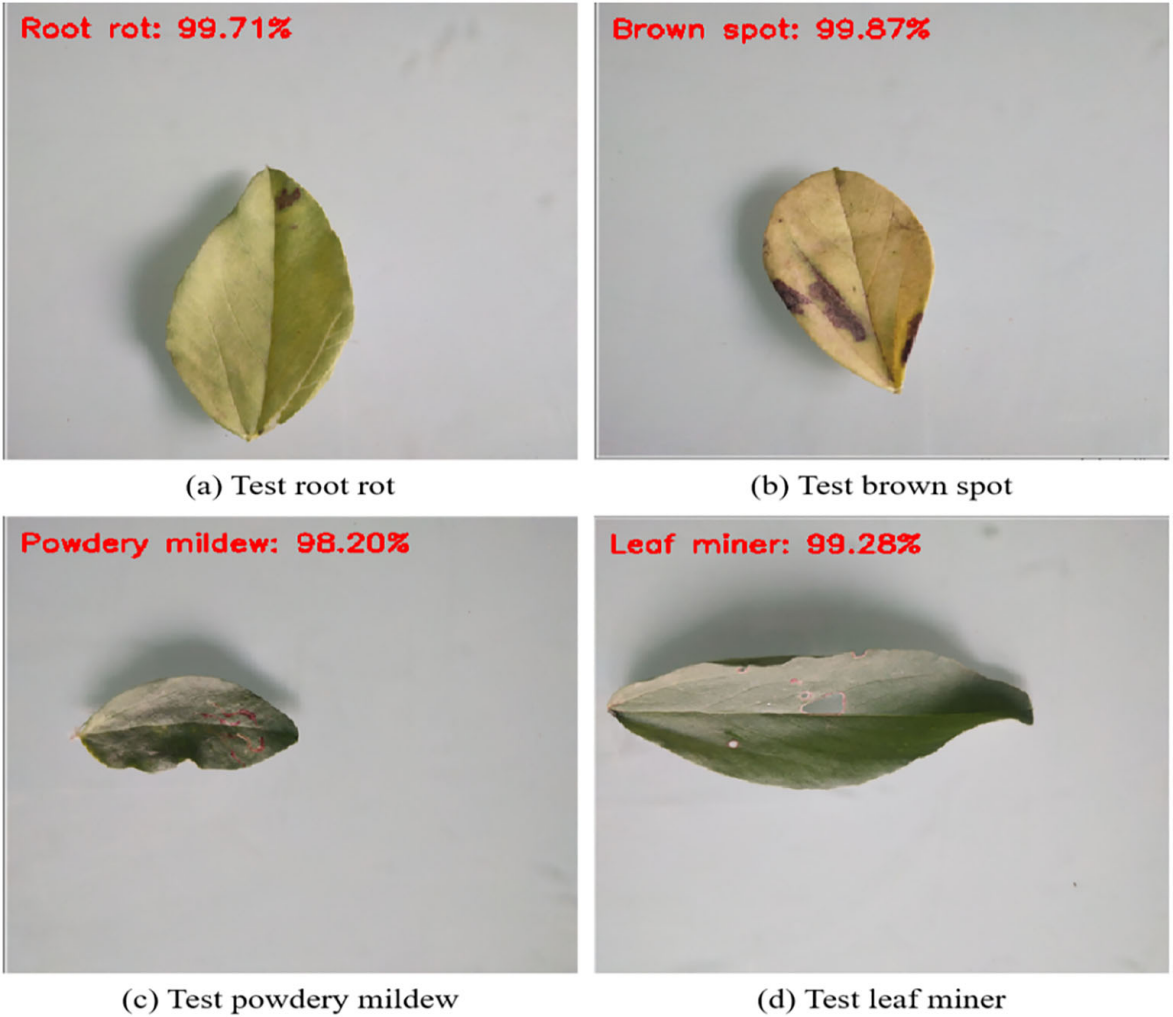
The testing results with the proposed model. It reflects the result of the proposed model randomly selecting a sample from the test set for prediction.

We used the suggested deep learning model to predict four different sets of selected samples from the test set. **Figure 12** displays visual prediction results. The category on the left represents the predicted category. The numerical value on the right represents the probability value of belonging to that category. Each recognition probability is greater than 90%.

**Figure 13:**
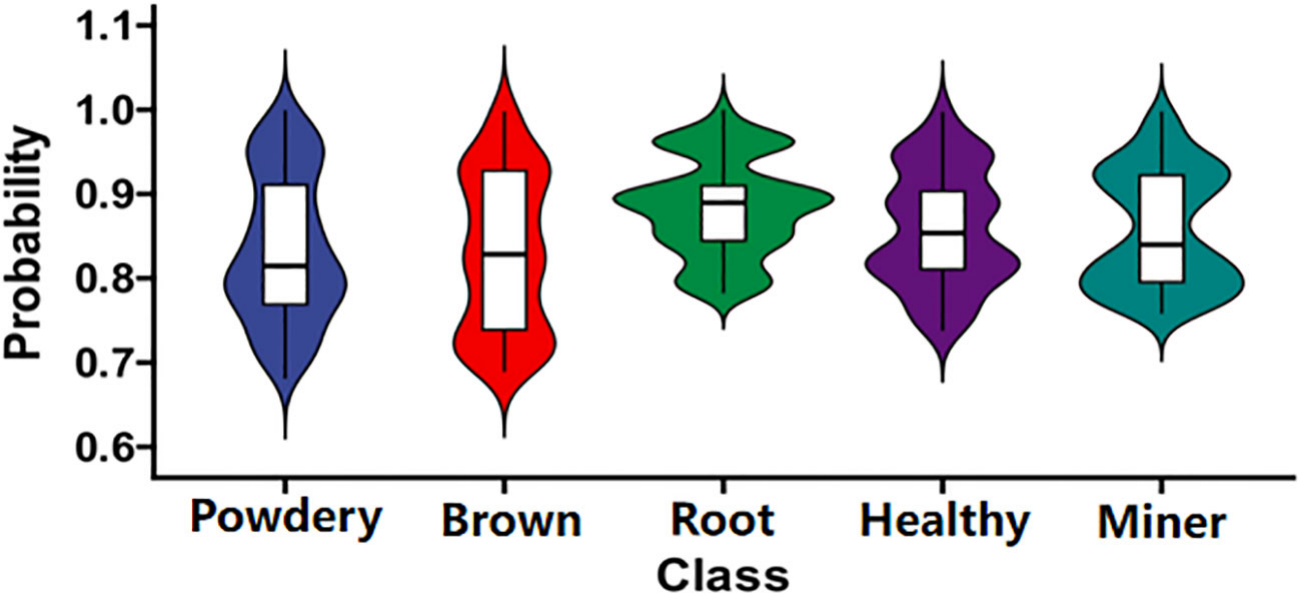
Identifying the correct probability distribution violin chart. It reflects the probability distribution of the proposed model for identifying five types of pea leaf diseases with correct categories.

To compare the overall probability values of the proposed approach for correctly identifying five types of pea leaf diseases for each category, we used the violin chart to present them in **Figure 13**.

As illustrated in **Figure 13**, the proposed model achieves a probability of over 60% in identifying the correct category of pea leaf diseases. Among the diseases, the root rot exhibits the best recognition performance, with the highest median probability and the most concentrated distribution of high probability values. In contrast, the probability distributions for the powdery mildew and brown spot diseases are more uneven. In summary, the proposed TSSC model demonstrates strong classification capability in identifying pea leaf diseases. These results also confirm the feasibility of integrating three attention mechanisms into a CNN architecture.

## 5 Discussion

In recent years, deep learning technology has become a key driving force for crop disease recognition. Especially, the Vision Transformer (ViT) and its variants have gradually overcome the limitations of classical CNN in global feature modeling through attention mechanisms, laying the foundation for the transition of agricultural production from labor-intensive manual detection to intelligent real-time monitoring. For example, Saleem et al. (2025) proposed a deep learning multi-scale feature processing method for extracting and combining plant leaf disease classifications, achieving a classification accuracy of 99.75% on datasets containing multiple diseases. Usman et al. (2025) proposed a method based on visual transformers for fusing spectral and spatial information in images with an accuracy of 98%. These studies indicate that ViT and its variants have significant advantages in global feature modeling, capturing cross-regional relationships, and lightweight deployment.

To further explore the performance boundaries of the proposed model, we compared the ViT-Base model with the proposed TSSC model. The comparison results are shown in **Table 3**.

**Table 3:** Experimental parameter configuration.

Models	Specificity	Precision	Recall	Accuracy
ViT-Base	0.9021	0.8954	0.9208	0.9186
Our model	0.9847	0.9975	0.9826	0.9952

**Table 3** reports that the proposed model outperforms ViT-Base across all four evaluation metrics. Although the ViT captures long-range dependencies through self-attention, CNN-based feature extraction-grounded in local receptive fields-proves more effective in tasks where fine-grained local features such as lesion edges and textures are critical for crop disease identification. Moreover, the introduced modules, including TNCA, CSE, and SA, compensate for the limitations of traditional CNNs in integrating global information, thereby contributing to the model’s superior performance.

From a broader literature perspective, the advantages of ViT and CNN models are task dependent. In tasks dominated by large-scale and long-distance correlations, such as monitoring the distribution of pests and diseases in panoramic farmland, ViT has a more significant advantage. For example. Wang et al. (2022) proposed a backbone network based on improved SwinT and applied it to data augmentation and recognition of actual cucumber leaf diseases, achieving a classification accuracy of 98.97%. However, in crop disease recognition tasks driven by local details, CNN models, especially those with attention enhancement, perform better. For example, on the open-source PaddyLeaf dataset, DenseNet achieved an accuracy of 99.21%. This pattern is consistent with our conclusion, further supporting that improving CNN is more suitable for visual tasks such as crop diseases, where local details are crucial.

However, the existing detection framework is only suitable for the identification of particular diseases in peas. It is unable to detect numerous illnesses on the same leaf. Furthermore, the framework has issues such as low sample size and poor coverage of disease categories, and it is influenced by factors such as climate, shooting angles, and disease severity, resulting in major limitations in recognition accuracy. Based on these limits, we created the following future work plan to clarify feasibility, probable hurdles, and predicted improvement directions.

### 5.1 Expand the multi-crop disease dataset

To efficiently collect multi-crop and multi-disease samples, we plan to leverage the growing adoption of hyperspectral imaging equipment and collaborate with agricultural research institutions. However, this endeavor faces several challenges. The fine-grained annotation of crop diseases relies heavily on expert knowledge, resulting in high labeling costs. Moreover, phenotypic variations of diseases across different crops may lead to domain shift, which could impair cross-crop recognition performance. As part of our future work, we aim to construct a multi-crop disease dataset comprising at least 10 crop species and 20 disease types, with no fewer than 1000 samples per disease. Domain adaptation methods will also be introduced to improve cross-crop recognition accuracy by over 15%.

### 5.2 Mobile deployment optimization

The mobile deployment of models is driven by improvements in edge computing capabilities and advances in model compression and mobile artificial intelligence frameworks. Nevertheless, several challenges remain. Lightweight models often suffer from reduced accuracy, while on-site factors such as lighting variations and physical vibrations can degrade image quality and inference reliability. To address these issues, we aim to compress the model to less than one-fifth of its original size via distillation and quantization, while limiting the accuracy drop to within 3%. Additionally, a real-time mobile image enhancement module will be integrated to reduce the false positive rate by 10% in challenging environments.

### 5.3 Apply hyperspectral imaging technology

The technology has established a solid foundation in plant disease detection. The commercial adoption of integrated solutions on portable devices and mobile platforms further facilitates large-scale sample collection. However, several challenges remain. For instance, processing high-dimensional and redundant hyperspectral data requires overcoming technical bottlenecks in fusing the hyperspectral and RGB imaging modalities. To address this, we plan to reduce the dimensionality of hyperspectral data to one-tenth of its original size using feature extraction algorithms and design a multimodal fusion network. This approach aims to enhance the accuracy of simultaneous multi-disease recognition compared to the RGB-only methods, ultimately achieving precise identification of multiple diseases on a single leaf.

## 6 Conclusions

In this work, we gathered five different pea leaf infectious diseases for the purpose of putting our proposed model through its paces. First, we used a three-neighbor channel attention module and squeeze-and-excitation network to extract features of healthy and diseased samples. Then, to achieve the accurate identification of pea leaf diseases, we combined the output features and sent them to a split attention network. We utilized the specificity, precision, recall, the f1-score, accuracy, and confusion matrices for comparisons with the representative classification algorithms, such as PNN, VGG-16, GoogLeNet, Inception-v4, and Efficient-B7. The findings provide conclusive evidence that the model that was proposed is superior. Furthermore, when evaluated on other standard datasets, the model attained an average accuracy of 99.41%, confirming its robust generalization capability.

In conclusion, our work has successfully created a novel deep learning system that can effectively detect pea leaf diseases with high accuracy. This breakthrough technology has the potential to significantly enhance the efficiency of image analysis for pea leaf diseases.

Although we have achieved high-precision classification and recognition of pea leaf diseases by integrating the TNCA, CSE, and SA modules, our work still has some limitations. In the future, we will deepen our research in the following directions to enhance the generalization ability, practical value, and robustness of the model.

### 6.1 Expansion of data dimensions

Current research has been limited to disease data from a single pea crop, leaving the model’s cross-crop generalization ability insufficiently validated. To address this, we plan to systematically expand the data dimension by collecting over 30 types of leaf disease samples from common crops such as wheat, rice, and tomato. This will enable the construction of a comprehensive multi-crop, multi-disease dataset. Furthermore, hyperspectral imaging technology will be introduced to acquire multimodal samples and enrich the feature information of diseases. These efforts will help validate the model’s adaptability in cross-crop scenarios and overcome the limitations of single-crop studies through multi-source data fusion, thereby laying the groundwork for the model’s broader application.

### 6.2 Implementation application promotion

We will focus on promoting the implementation and application of the model. To this end, the model is optimized using techniques such as knowledge distillation and quantization pruning to make it suitable for deployment on terminals like smartphones and portable edge devices. Concurrently, a dedicated mobile application has been developed to port the optimized model onto devices. Field experiments were conducted in real pea planting environments under diverse conditions—including varying weather (e.g., rainy and sunny), lighting ((e.g., morning and evening), and shooting angles-to validate the model’s real-time performance and stability in complex scenarios. These efforts aim to establish an integrated field inspection solution that enables “image acquisition, real-time recognition, and result feedback,” thereby bridging the gap between theoretical research and agricultural practice.

### 6.3 Technological depth extension

For disease types that exhibit high similarity in small lesion features, we will optimize the feature extraction procedures within the TNCA, CSE, and SA attention modules to enhance their capacity to capture discriminative disease characteristics. Furthermore, to mitigate common challenges in field imaging—such as lighting noise, image blur, and background interference—we will incorporate adversarial training and adaptive image enhancement techniques. These improvements are designed to strengthen the model’s robustness to noisy inputs and ensure reliable recognition performance in complex field environments.

### 6.4 Hyperspectral imaging technology

We will use hyperspectral imaging technology to enhance the identification of plant leaf diseases. Hyperspectral imaging technology can capture spectral changes related to plant physiological changes. The increasing popularity of portable hyperspectral devices and integrated mobile solutions, in particular, further supports efficient large-scale data collection under on-site conditions. We plan to develop a dedicated processing framework to address the complexity of processing hyperspectral data due to its high dimensionality and inherent redundancy. This framework combines advanced feature dimensionality reduction algorithms to compress hyperspectral data into smaller sizes. We will implement a cross-modal fusion network to align and combine spectral and spatial features of hyperspectral and RGB sources and achieve the reliable multi-disease detection capability.

## Data Availability

The original contributions presented in the study are included in the article/supplementary material. Further inquiries can be directed to the corresponding authors.
